# Primary Biliary Cholangitis in the Middle East: A Review of Current Challenges and Treatment Approaches

**DOI:** 10.7759/cureus.113526

**Published:** 2026-07-28

**Authors:** Faisal M Sanai, Abdullah S Alghamdi, Abdullah A Khathlan, Adel Alqutub, Huda Al Quraishi, Mohammed Y Alghamdi, Mohamed E Alzaabi, Mohammad H Mawardi, Munira Y Al-Tarrah, Nawal AlNahdi, Ahmad Al-Rifai

**Affiliations:** 1 Department of Medicine, Gastroenterology Section, King Abdulaziz Medical City, Ministry of National Guard Health Affairs, Jeddah, SAU; 2 Department of Medicine, Gastroenterology Unit, King Fahad Hospital Jeddah, Jeddah, SAU; 3 Department of Gastroenterology and Hepatology, King Fahad Medical City, Riyadh, SAU; 4 Department of Gastroenterology and Hepatology, Rashid Hospital, Dubai Health, Dubai, ARE; 5 Department of Internal Medicine, Gastroenterology, Hepatology and Liver Transplant, King Fahd Military Medical Complex, Dhahran, SAU; 6 Department of Gastroenterology and Hepatology, Zayed Military Hospital, Abu Dhabi, ARE; 7 Department of Gastroenterology and Hepatology, Sheikh Khalifa Medical City, Abu Dhabi, ARE; 8 Department of Medicine, Gastroenterology Section, King Faisal Specialist Hospital and Research Center, Jeddah, SAU; 9 Department of Internal Medicine, Al-Amiri Hospital, Kuwait, KWT; 10 Department of Gastroenterology, Hepatology and Endoscopy, Mohammed Bin Rashid University of Medicine and Health Sciences, Dubai Health, Dubai, ARE; 11 Department of Medicine, Sheikh Shakhbout Medical City, Abu Dhabi, ARE

**Keywords:** epidemiology, gulf region, pparδ agonists, primary biliary cholangitis, second-line therapy

## Abstract

Primary biliary cholangitis (PBC) is a chronic autoimmune cholestatic liver disease that can progress to fibrosis, cirrhosis, and liver failure. Most evidence guiding diagnosis and management is derived from Western populations, while Middle East-specific data remain limited. This review summarizes global and regional evidence and identifies Gulf-specific challenges affecting PBC recognition and care. A literature search of PubMed and Embase (2014-2025) was conducted to identify studies addressing PBC epidemiology and management in the Middle East. In parallel, expert insights were collected from 11 Gulf-based hepatologists through pre-meeting questionnaires and structured discussions. A separate online physician survey assessed awareness, diagnostic pathways, treatment access, and perceived barriers across the Gulf region. Published regional data were sparse and largely limited to small, single-center cohorts, preventing reliable population-level estimates. Experts perceived PBC as underdiagnosed and inconsistently referred, with variable access to confirmatory autoantibody testing and specialist hepatopathology. Treatment was based on ursodeoxycholic acid (UDCA), while access to second-line therapies remained inconsistent despite the substantial proportion of incomplete UDCA responders reported in international literature and guidelines. Emerging agents have demonstrated meaningful biochemical and symptom benefits in phase 3 studies, highlighting a growing gap between evolving evidence and regional therapeutic availability. Overall, PBC in the Middle East remains under-characterized and likely under-recognized. Establishing regional registries, standardizing referral and diagnostic pathways, and improving access to evidence-based second-line therapies should be prioritized to optimize patient outcomes.

## Introduction and background

Primary biliary cholangitis (PBC) is a chronic, progressive cholestatic liver disease characterized by immune-mediated destruction of the small intrahepatic bile ducts, leading to fibrosis, cirrhosis, and, ultimately, liver failure [1]. PBC predominantly affects middle-aged women and is frequently associated with other autoimmune diseases [2]. Its pathogenesis involves a complex interplay of genetic predisposition, environmental triggers, and immune dysregulation [3]. A hallmark of the disease is the presence of anti-mitochondrial antibodies (AMA), along with elevated serum alkaline phosphatase (ALP) levels, which are central to establishing the diagnosis [2].

The current first-line treatment for PBC is ursodeoxycholic acid (UDCA) [4], which improves liver biochemistry and slows disease progression, especially when started early [5]; however, 25-50% of patients are incomplete responders, and second-line options such as obeticholic acid (OCA), fibrates, and peroxisome proliferator-activated receptor (PPAR)-targeted agents are increasingly used in these patients [6,7]. Some patients nonetheless progress to end-stage liver disease requiring transplantation [8], though emerging therapies continue to expand the treatment landscape [9]. Globally, PBC incidence and prevalence vary by region, with higher rates in North America and Northern Europe than in Asia, reflecting genetic, environmental, and healthcare-related factors [1,10]. In contrast, PBC remains underrecognized and underreported in the Middle East, particularly within the Gulf Cooperation Council (GCC) countries, and limited region-specific data obscure potential differences in phenotype, diagnostic pathways, and treatment access [10,11].

**Figure 1:**
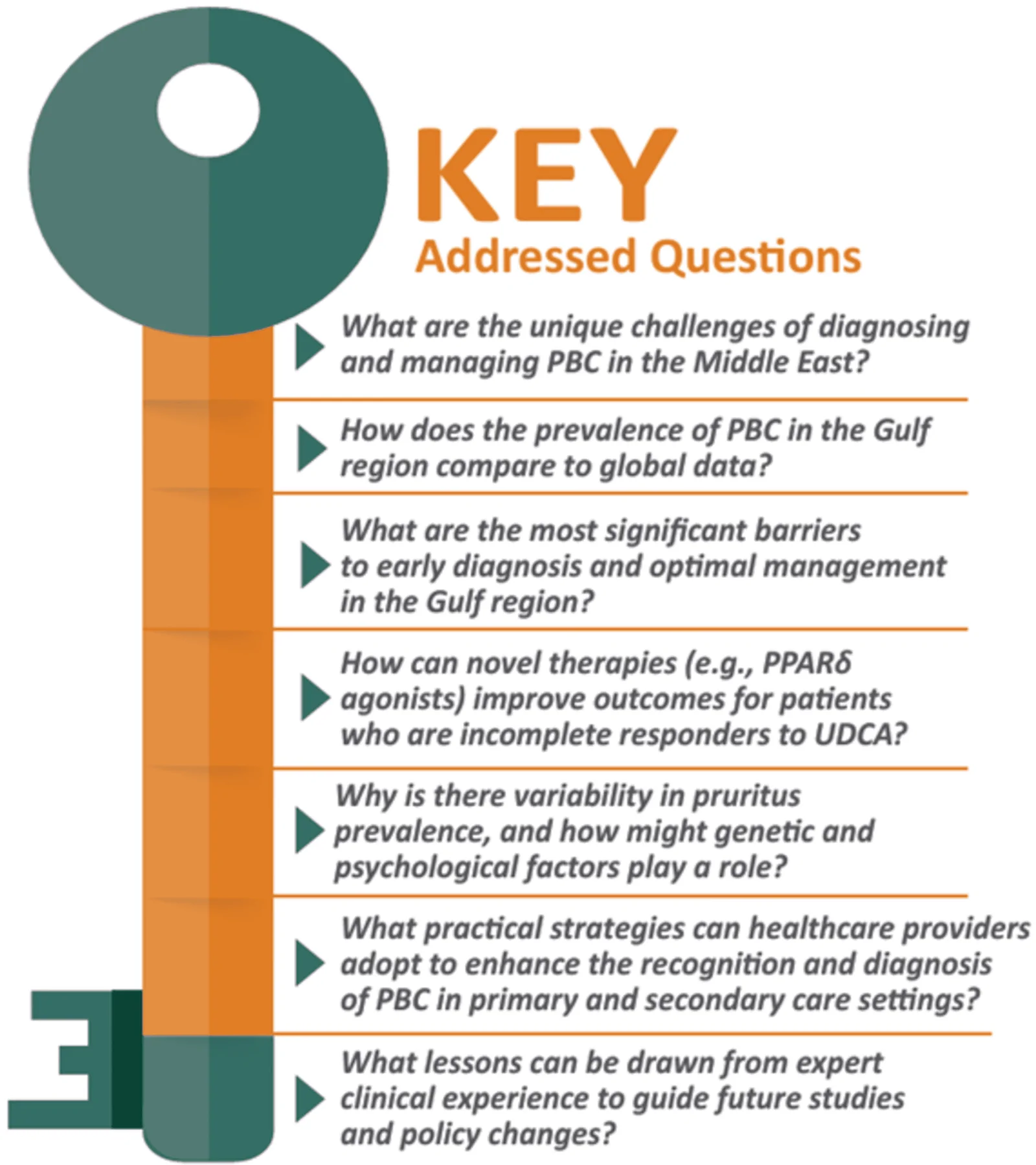
Summary of key addressed questions. PBC: primary biliary cholangitis. Image created by authors using Adobe Illustrator 2026 (Adobe Inc., San Jose, CA, USA).

This article synthesizes available global and regional evidence on PBC and integrates insights from Gulf-based hepatologists to characterize the epidemiology, diagnostic challenges, and management landscape of PBC in the Middle East. In parallel, findings from a structured regional physician survey provide additional perspectives on real-world practice patterns and unmet needs. A summary of the key questions addressed is presented in Figure 1.

Methods

A comprehensive literature search was conducted in PubMed and Embase for English-language publications between 2014 and 2025 addressing PBC epidemiology and management, with a focus on Middle Eastern populations. In parallel, expert opinion was collected through a structured pre-meeting questionnaire and a moderated discussion involving 11 hepatologists practicing across the Gulf region. Expert insights were synthesized narratively to identify common challenges and areas of consensus. This approach was intended to contextualize the published literature with regional clinical experience, rather than to generate primary research data. This review is a hybrid narrative review that integrates a structured literature search, expert consensus derived from an 11-member hepatologist panel, and a physician survey; it is not a systematic or scoping review, and no formal quality appraisal of included studies was performed. Throughout this review, “Middle East” refers broadly to the geographic and literature scope, “Gulf region” and “GCC countries” refer specifically to the six Gulf Cooperation Council states represented by the expert panel and survey, and “Saudi Arabia” is used only when referring to country-specific data.

A structured online physician survey (SurveyMonkey, San Mateo, CA) was administered to hepatologists and gastroenterologists practicing across the Gulf region to assess awareness, diagnostic pathways, treatment access, and perceived barriers to PBC care. The 30-item instrument covered three domains: (i) respondent demographics and practice characteristics (specialty, years in clinical practice, monthly chronic liver disease caseload, practice setting, and country of practice); (ii) diagnostic awareness and practice patterns (familiarity with PBC, perceived local prevalence, recognition of typical clinical features, the ALP threshold used to trigger further testing, investigations ordered for unexplained cholestasis, confidence distinguishing PBC from clinical mimics, and referral behavior and barriers); and (iii) treatment patterns and educational needs (frequency of complete biochemical response to UDCA, second-line agents used, awareness of emerging therapies, prior continuing medical education (CME) exposure, and preferred formats for future education). Eleven physicians completed the survey, practicing in Saudi Arabia (n=5, 45%), Kuwait (n=5, 45%), and the United Arab Emirates (n=1, 9%); most identified as hepatologists (n=6, 55%) or gastroenterologists (n=4, 36%), with one internal medicine physician (9%) and three respondents specifying additional subspecialty training (transplant hepatology, hepatology/endoscopy, and infectious disease). The majority had more than 20 years of clinical experience (n=6, 55%) and managed more than 20 patients with chronic liver disease per month (n=8, 73%), practicing predominantly in public hospital settings (n=9, 82%), with some also affiliated with private clinics (n=3, 27%) or liver transplant centers (n=3, 27%). The survey was distributed to 11 physicians, all of whom responded, yielding a response rate of 100% (11/11); however, only eight of the 11 respondents (73%) completed the full instrument beyond the demographic section, corresponding to a completion rate of 73%. Responses were summarized descriptively as frequencies and percentages using SurveyMonkey’s native analytics; given the small, non-random sample, survey findings are considered exploratory and hypothesis-generating rather than representative of regional practice, and are reported alongside, but distinguished from, the expert panel discussion.

## Review

Epidemiology

Globally, the pooled prevalence and incidence of PBC are estimated to be approximately 14.6 and 1.8 per 100,000 persons, respectively, based on a meta-analysis by Lv et al. [10], with higher rates reported in studies published after 2010. Substantial regional variability has been consistently observed. In Europe, a systematic review by Gazda et al. reported a pooled point prevalence of 22.27 per 100,000 inhabitants [12]. Notably, marked heterogeneity exists within Europe, with northern and northwestern populations presenting with more advanced biochemical disease and experiencing poorer outcomes, including higher rates of decompensation and reduced transplant-free survival, compared with southern European populations [13]. These differences are likely multifactorial, reflecting interactions between genetic susceptibility, environmental exposures, healthcare access, and disease phenotype [13,14].

In North America, prevalence estimates vary among studies due to differences in study design and populations [15]. A multicenter study from the Fibrotic Liver Disease Consortium reported an overall 12-year period prevalence of 29.3 per 100,000 persons across multiple U.S. health systems, based on diagnosed cases recorded in healthcare registries, with notable regional variation [16,17].

Historically, epidemiological data from the Asia-Pacific region were limited; however, recent meta-analyses indicate that both incidence and prevalence are higher than previously assumed [18]. Between 2009 and 2019, prevalence varied across subregions, with higher rates reported in East Asia (15.6 per 100,000) and lower rates observed in Oceania (approximately 5.3 per 100,000) [19].

In contrast to this extensive data from Western populations, the epidemiology of PBC in the Middle East and Gulf region remains poorly defined [11,18,20]. A focused literature search identified only a small number of studies, primarily from Saudi Arabia and Iran. A retrospective cohort study from Riyadh evaluated 30 patients diagnosed between 2008 and 2017, with a primary focus on biochemical and clinical responses to UDCA rather than on prevalence estimation [20]. The low number of cases reported may suggest either a low prevalence of PBC in Saudi Arabia or the possibility of underdiagnosis or referral bias. It is also plausible that cases are being managed locally without referral to specialized tertiary centers [20]. Similarly, a cohort study from Iran evaluating autoimmune liver diseases reported PBC in 14% of patients but did not provide population-based prevalence estimates. While this study also did not specifically target epidemiology, it provided valuable insights into clinical features and treatment responses in PBC patients [21].

Despite these limitations, progress toward standardizing PBC care in the region has been made. The publication of national guidelines by the Saudi Association for the Study of Liver Diseases and Transplantation (SASLT) in 2021 represents a key milestone, providing contextually adapted recommendations for cholestatic liver diseases, including PBC [11].

Nevertheless, in the absence of population-based epidemiological studies, the true prevalence and distribution of PBC in the Middle East remain unknown. Establishing a national disease registry and conducting well-designed epidemiologic research are essential next steps to inform healthcare planning, resource allocation, and policy development across the region.

Expert Opinion on Epidemiology

The epidemiology of PBC in the Gulf region remains poorly defined due to the absence of population-based studies and national disease registries. Based on insights from 11 hepatologists actively managing PBC across the region, the disease is generally perceived as less prevalent than in Western populations, with substantial variability reported between countries and healthcare centers.

**Figure 2:**
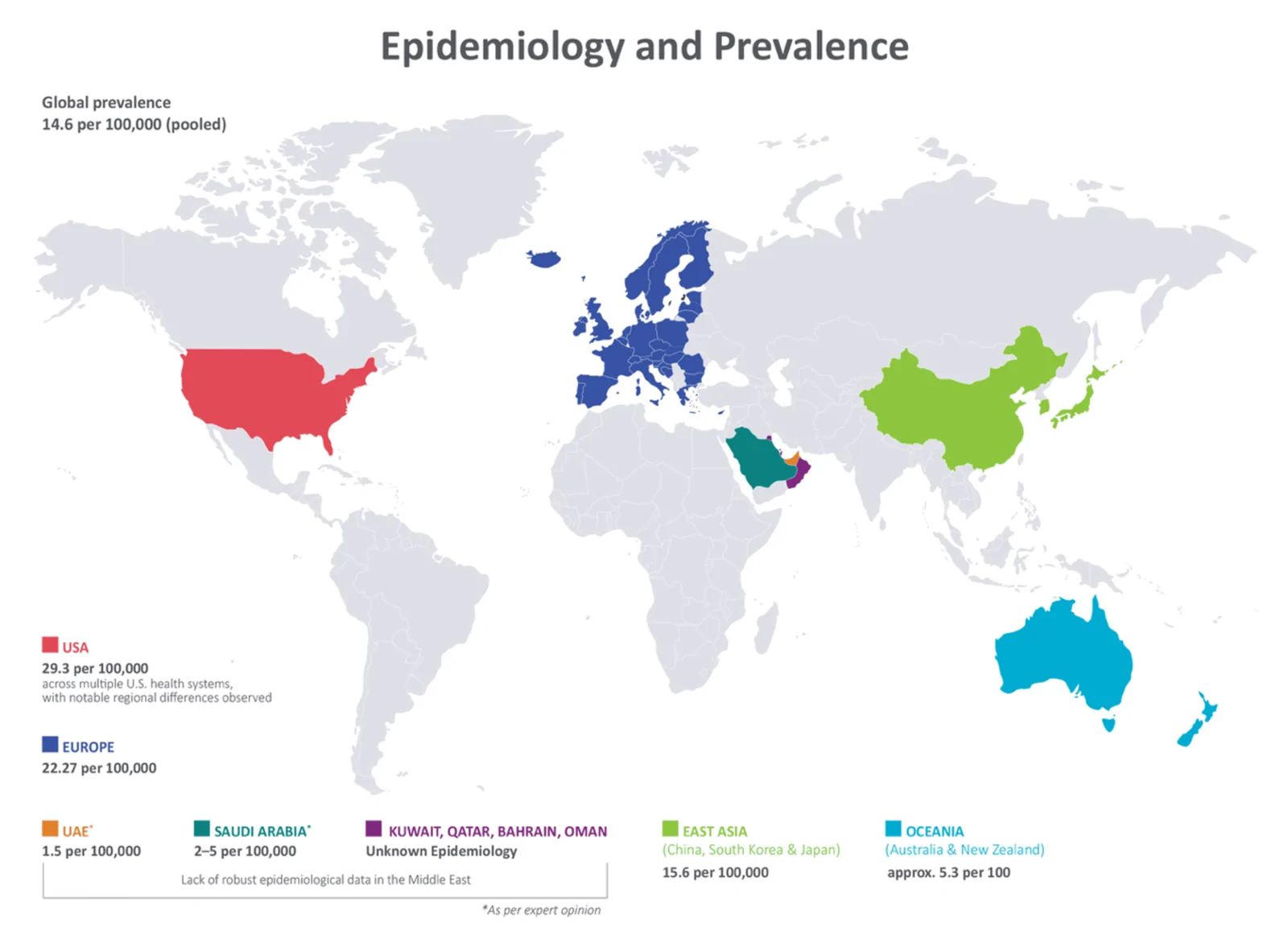
Published and expert-perceived PBC prevalence. Note: Estimates for the USA, Europe, East Asia, and Oceania are derived from published, population-based epidemiologic studies; estimates for Saudi Arabia and the UAE reflect expert opinion only and are not derived from population-based studies. PBC: primary biliary cholangitis. Image created by authors using Adobe Illustrator 2026 (Adobe Inc., San Jose, CA, USA).

Based on panel consensus, no single hospital among those represented appears to manage a very large volume of PBC patients; however, several tertiary centers in Saudi Arabia function as regional referral hubs. High-volume centers typically follow 20-50 cumulative PBC cases, while many institutions report considerably fewer patients. These expert opinion figures are hypothesis-generating only and are not equivalent to population-based epidemiologic estimates; they should not be interpreted as validated prevalence data. Based on expert estimates, the prevalence of PBC in Saudi Arabia was approximated at 2-5 per 100,000, corresponding to 500-1,500 patients nationwide. In the United Arab Emirates, particularly in Dubai, prevalence was estimated at approximately 1.5 per 100,000, translating to 200-300 patients in a population of around 3.5 million. These figures should be interpreted cautiously, as they are derived from clinical experience rather than formal epidemiologic studies, and may reflect both underdiagnosis and referral bias. Notably, no published prevalence data are currently available from Kuwait, Qatar, Bahrain, or Oman. A visual representation of global PBC prevalence is shown in Figure 2.

Compared with global estimates, the Gulf region appears to have a lower reported prevalence of PBC. Experts attributed this discrepancy primarily to under-recognition of the disease, limited access to specialized hepatology services, inconsistent referral pathways, and the tendency for milder cases to be managed in primary or non-specialist settings.

A distinctive feature of PBC in the Gulf region is the relatively high frequency of overlap syndromes, often confirmed histologically. Experts estimated that approximately 70% of patients present with pure PBC, while the remainder exhibit overlap features, most commonly with autoimmune hepatitis or other cholestatic liver diseases. In addition, based on panel consensus rather than published regional data, a substantial proportion of patients are AMA-negative, posing diagnostic challenges and frequently necessitating liver biopsy, particularly when treatment is initiated prior to definitive histological confirmation.

Taken together, these findings highlight the urgent need for structured epidemiological studies to accurately define the burden of PBC in the Gulf region. Establishing multicenter registries, improving disease surveillance, and standardizing data collection through collaborative research efforts are essential to address existing knowledge gaps and optimize patient care.

Diagnosis and current treatment landscapes

For practical application in the Middle East/Gulf context, the diagnostic and treatment-response principles described above can be summarized as a stepwise algorithm: (1) evaluate cholestatic liver enzymes (ALP, GGT) [22,23]; (2) test for AMA/AMA-M2 [22,23]; (3) if AMA-negative but PBC is still suspected, test for anti-gp210/anti-Sp100 [24]; (4) consider abdominal ultrasound or magnetic resonance cholangiopancreatography (MRCP) where biliary obstruction or an alternative diagnosis is possible--reflecting standard clinical practice rather than a specific citation within this review; (5) consider liver biopsy when serology is inconclusive, when an overlap syndrome is suspected, or when AMA is negative [20,25]; (6) initiate UDCA and formally assess biochemical response at 12 months using validated criteria [6,26]; and (7) refer for second-line therapy or specialist hepatology input if the response is inadequate [27].

The diagnosis of PBC follows a structured approach, particularly in patients presenting with cholestatic liver disease enzyme abnormalities [11]. Persistent elevation of alkaline phosphatase (ALP) ≥1.5× the upper limit of normal (ULN) for more than 24 weeks, in the presence of AMA, especially the AMA-M2 subtype at a titer ≥1:80, remains a cornerstone of diagnosis and forms the basis of widely adopted diagnostic algorithms [22,23]. Per European Association for the Study of the Liver (EASL)/American Association for the Study of Liver Diseases (AASLD) guidance, a confident diagnosis of PBC requires at least two of the following three criteria: biochemical evidence of cholestasis (elevated ALP), a positive AMA (or a PBC-specific ANA pattern in AMA-negative cases), and/or compatible histology; AMA positivity is therefore supportive of, but not strictly mandatory for, the diagnosis [4,28]. Additional elevations in gamma-glutamyl transferase (GGT) and IgM are frequently observed; IgM is elevated in up to 80% of patients and may correlate with disease activity, although it is not universally used in isolation for clinical decision-making [24,29].

Most patients are asymptomatic or have mild biochemical abnormalities at presentation; however, approximately 5-10% present with icteric cholestasis. This presentation is often associated with the premature ductopenic variant of PBC, characterized by rapid bile duct loss, marked jaundice, hyperlipidemia, and progression to cirrhosis or liver failure within five years [30]. Such patients are typically identified incidentally, most often through elevated ALP detected during routine blood testing, pre-operative screening, or evaluation for other autoimmune conditions, rather than through symptom-triggered presentation [30]; this pattern of incidental detection underlies much of the diagnostic delay discussed later in this review.

Non-invasive markers, such as ALP and bilirubin, are important for monitoring disease progression and predicting outcomes in PBC, including liver transplantation and mortality [11]. However, AMA is present in approximately 90-95% of patients with PBC and confers high diagnostic specificity, targeting mitochondrial 2-oxo-acid dehydrogenase complexes [4,15]. In the minority of AMA-negative patients (5-10%), misdiagnosis may occur, and additional serologic markers, such as anti-Sp100 and anti-gp210, are valuable for diagnostic confirmation [24]. However, access to these extended antibody panels varies widely across centers. In the only published Middle Eastern cohort to date, AMA positivity was reported in 86.7% of patients from a Saudi Arabian tertiary center, consistent with global data and reinforcing the diagnostic reliability of AMA in regional populations [20].

Although not essential for establishing a diagnosis, a liver biopsy may be considered in cases of inconclusive test results or to assess disease severity [25]. AMA-negative presentations necessitate liver biopsy to establish a definitive diagnosis and differentiate PBC from overlapping or mimicking hepatobiliary conditions [20]. Histologically, PBC is characterized by chronic cholangitis involving interlobular and septal bile ducts, with distinctive features such as florid duct lesions and epithelioid granulomas in the early stages. The disease progresses through four stages, ranging from portal inflammation (Stage I) to cirrhosis with regenerative nodules (Stage IV). Due to the patchy nature of lesions, obtaining an adequate tissue sample with at least 10-15 portal tracts is crucial [28].

Transient elastography (TE) has increasingly been adopted as a non-invasive tool for assessing liver stiffness and fibrosis severity in PBC [31]. TE provides quantitative, repeatable measurements that correlate with fibrosis stage and cirrhosis risk, supporting both baseline assessment and longitudinal disease monitoring, while avoiding the risks associated with invasive biopsy [32,33].

Advances in first- and second-line therapies have improved PBC management by slowing disease progression and improving quality of life, yet unmet needs persist, particularly among patients who do not respond adequately to standard treatment.

First-Line Treatment: Ursodeoxycholic Acid (UDCA)

UDCA remains the first-line therapy and has demonstrated efficacy in normalizing liver biochemistry, delaying histological progression, and improving transplant-free survival [1]. Its therapeutic effects are mediated through improved bile acid flow, reduced bile toxicity, and anti-inflammatory and anti-fibrotic mechanisms [34]. The EASL guidelines (2017) recommend UDCA at a dose of 13-15 mg/kg/day as first-line therapy for all patients with PBC, including those with advanced disease stages [4]. Reductions in ALP and bilirubin under UDCA therapy are strongly associated with improved long-term outcomes [35].

While UDCA is the standard of care, up to 40% of patients may not achieve an adequate response [26]. A recent large registry-based study by Cortez-Pinto et al. (2021) showed that predictors of incomplete response to UDCA include older age at diagnosis, high baseline ALP levels, and the presence of cirrhosis [6]. In such patients, continued UDCA monotherapy may be insufficient to halt disease progression, necessitating consideration of second-line therapies.

Incomplete (or non-) response to UDCA is generally defined using validated biochemical criteria, such as the Paris I/II, Barcelona, Rotterdam, or Toronto criteria, based on thresholds for ALP, bilirubin, and/or aspartate aminotransferase (AST) measured after 12 months of therapy [6,26]. In the Gulf region, UDCA is universally used as first-line therapy and is generally effective in early-stage disease. However, regional data highlight important limitations. A retrospective cohort from Riyadh demonstrated substantial variability in biochemical response depending on the criteria applied: 73.3% met Paris I response criteria, compared with 40.0%, 56.7%, and 53.3% under Paris II, Barcelona, and Toronto criteria, respectively [20]. Overall, between 27% and 60% of patients failed to meet response thresholds, underscoring the unmet need for alternative or adjunctive therapies in this setting. Interpretation of these findings is limited by the small sample size, highlighting the need for larger regional studies [20].

Second-Line Treatments

Obeticholic acid (OCA), a farnesoid X receptor (FXR) agonist, was for several years the principal approved second-line therapy for patients with an inadequate response to UDCA [27]. By modulating bile acid synthesis and transport, OCA reduces cholestasis and improves surrogate biochemical markers, including alkaline phosphatase [36,37]. However, its clinical use is limited by adverse effects, most notably pruritus, and safety concerns in patients with advanced liver disease [38]. In 2021, the FDA issued a boxed warning advising against OCA use in patients with advanced cirrhosis (Child-Pugh B or C) following post-marketing reports of hepatic decompensation and liver failure [39]. The regulatory landscape for OCA has since changed substantially: the European Commission revoked Ocaliva's marketing authorization for PBC in 2024 after the COBALT trial failed to demonstrate a survival benefit in the primary intent-to-treat analysis [40]; the FDA declined to convert OCA's accelerated approval to full approval, issuing a Complete Response Letter on November 12, 2024 [41]; and in September 2025, Intercept Pharmaceuticals voluntarily withdrew OCA from the U.S. market at the FDA's request, with the withdrawal taking effect in November 2025 and all US clinical trials involving OCA placed on clinical hold [42].

Since 2024, two additional agents have received U.S. FDA accelerated approval as second-line options for UDCA non-responders or UDCA-intolerant patients, meaningfully expanding the treatment landscape beyond OCA. Among these, seladelpar (Livdelzi), a highly selective PPAR-δ agonist that modulates bile acid metabolism, received FDA accelerated approval in August 2024 [43] and has demonstrated the most promising profile to date [27]. In the RESPONSE Phase 3 trial, a 12-month, double-blind, placebo-controlled study involving patients with PBC who had an inadequate response to UDCA, 61.7% of patients receiving seladelpar achieved the primary composite biochemical response, compared to 20.0% in the placebo group [44]. Seladelpar was also associated with significant reductions in pruritus and sustained improvements in ALP, GGT, and bilirubin, without exacerbating itch or raising hepatotoxicity concerns, supporting its dual role in disease control and symptom relief [44].

Elafibranor, a dual PPAR-α/δ agonist, received FDA accelerated approval in June 2024 [45] and also demonstrated favorable biochemical responses in the ELATIVE Phase 3 trial, where 51% of treated patients achieved the composite biochemical response compared to 4% in the placebo group. While elafibranor did not significantly reduce pruritus severity on the Worst Itch Numeric Rating Scale (WI-NRS) [46], modest improvements were observed in patient-reported itch-related outcomes using the PBC-40 and 5-D Itch scales [47-49].

Fibrates, including bezafibrate and fenofibrate, are used off-label as adjunctive therapy to UDCA and have shown efficacy in reducing cholestatic markers such as ALP and GGT, notably in the BEZURSO trial [50]. However, concerns regarding long-term safety, particularly in patients with renal impairment, continue to limit their widespread adoption [51].

Linerixibat, an ileal bile acid transporter (IBAT) inhibitor, received FDA approval in March 2026 as the first medicine specifically indicated for cholestatic pruritus in adult patients with PBC, based on the GLISTEN Phase 3 trial. While it improves itch severity and quality of life, it does not modify biochemical disease progression and is therefore considered symptomatic rather than disease-modifying therapy [52].

Despite these expanding global approvals, access to second-line and emerging therapies in the Middle East remains limited, with UDCA often being the only routinely available option. Increasing clinician awareness and facilitating regulatory and formulary access to evidence-based therapies are essential steps toward improving outcomes for patients with PBC in the region.

Key insights from the cohort of hepatologists on the management of primary biliary cholangitis (PBC) in the Middle East

Pruritus in Primary Biliary Cholangitis (PBC): A Complex Symptom With Variable Prevalence

Pruritus is a hallmark symptom of PBC, with marked variability in prevalence across the region. While some centers report pruritus in fewer than 20% of patients, others observe it in more than 70%. This wide range likely reflects not only true differences in symptom burden but also heterogeneity in documentation practices, patient reporting, clinician awareness, and disease stage at diagnosis. Consistent with regional observations, published studies report pruritus prevalence ranging from 7% to 80%, influenced by study design, patient populations, and potential under-reporting [53,54].

Expert consensus suggests that pruritus is more frequently reported in earlier stages of PBC, often in association with fatigue, and tends to diminish as cirrhosis develops. Although pruritus can occur at any stage and may precede diagnosis by months or years, its attenuation in advanced disease suggests that severity of liver dysfunction influences symptom expression [55,56].

The pathogenesis of pruritus in PBC is multifactorial, involving hepatic and neuroimmune mechanisms. Cholestasis leads to accumulation of pruritogenic mediators, including autotaxin and lysophosphatidic acid, which activate sensory neurons directly or through immune-mediated pathways [57]. Cytokines such as interleukin-31 (IL-31) further amplify itch signaling via peripheral nerve receptors and central transmission through the spinothalamic tract [58]. The result is the conscious perception of itch [59]. Clinically, chronic pruritus significantly impairs quality of life, contributing to sleep disturbance, fatigue, and psychological distress [57].

Compared with Europe, where most patients with PBC report pruritus, prevalence appears lower in the Middle East [60]. This raises a hypothesis about a potential role of genetic differences, which warrants further investigation. Additionally, experts suggested that patient perception may play a role in the reporting of pruritus, though this remains unconfirmed. Repeated inquiries about the symptom may lead to heightened awareness, potentially triggering unconscious reporting of itching, highlighting the psychological influence on symptom perception. These genetic and psychological explanations are speculative hypotheses that have not been formally tested in the region and should not be interpreted as established mechanisms.

Management Strategies for Pruritus

Although UDCA remains the cornerstone of disease-modifying therapy in PBC, it does not reliably improve pruritus and may, in some cases, exacerbate symptoms [55]. First-line symptomatic treatment consists of bile acid sequestrants, including cholestyramine, colestipol, or colesevelam, which reduce systemic levels of pruritogens by binding bile acids in the intestine [56,61]. However, their disagreeable taste and gastrointestinal side effects, such as bloating and constipation, often limit adherence [55].

For patients with inadequate response after a short trial of bile acid sequestrants, rifampicin is recommended as second-line therapy and is effective in approximately 70% of cases by enhancing hepatic metabolism and clearance of pruritogens [55,61]. Third-line options include µ-opioid receptor antagonists, which mitigate cholestatic pruritus by counteracting elevated endogenous opioid activity [28].

Experts advocate a stepwise approach that combines pharmacological therapy with lifestyle interventions. Patients should be advised to avoid exacerbating factors such as heat, stress, and irritant fabrics, and to use emollients to maintain skin hydration. Antihistamines and topical agents may offer limited adjunctive benefit, particularly for nocturnal symptoms, but are not considered primary therapies. Despite its demonstrated efficacy, rifampicin remains underutilized in the region, partly due to limited availability and concerns regarding safety monitoring.

Emerging therapies, particularly PPAR-δ agonists, offer a promising approach to pruritus management while simultaneously improving cholestasis and fibrosis. Their antipruritic effects are thought to result from improved bile acid homeostasis and reduced hepatic inflammation, mediated in part through BCL6 pathways and downregulation of cytokines such as IL-31 [57,62]. Ongoing clinical trials are expected to clarify their therapeutic role and long-term safety profile.

Overall, effective management of pruritus in PBC requires a multifaceted strategy integrating lifestyle modification, stepwise pharmacologic therapy, and emerging disease-modifying agents to optimize symptom control and quality of life.

Delays in Diagnosis

Delayed diagnosis of PBC remains a major challenge in the Middle East and is largely driven by limited awareness among non-specialist physicians, delayed referral, and inconsistent interpretation of cholestatic liver enzymes. These delays reduce opportunities for early UDCA initiation, which is associated with improved biochemical response and long-term transplant-free survival. Inadequate inclusion or interpretation of ALP and GGT in routine liver function testing further contributes to underdiagnosis.

Improving awareness among frontline healthcare providers, particularly general practitioners, is essential. While gastroenterologists generally refer complex or treatment-refractory cases to hepatologists, increased recognition of abnormal liver enzymes by other specialties, such as dermatology, endocrinology, and rheumatology, has begun to improve referral patterns.

Autoantibody Testing: Inconsistencies Across Centers

While the introduction of an updated autoantibody testing panel in some centers has improved diagnostic accuracy, access to these tests remains inconsistent across the region. AMA-M2 testing is particularly valuable in supporting the diagnosis of PBC, yet many institutions lack local testing capabilities and rely on external laboratories. Moreover, experts highlighted the challenge of antibody titer reporting, as some laboratories provide only a binary positive/negative result rather than specific titer values, making interpretation difficult. The experts emphasized that ascertaining the titer level is crucial, as low-titer positivity may lack clinical significance, while high titers (≥1:80) strongly support a PBC diagnosis.

In cases where AMA-M2 is negative but PBC is still suspected, additional serological tests, such as anti-Sp100 and anti-gp210 antibodies, PBC-specific antinuclear antibody (ANA) patterns typically detected by indirect immunofluorescence or ELISA [24], or liver biopsy may be required for confirmation. However, access to reliable testing for antibodies, such as anti-Sp100 and anti-gp210, varies substantially, limiting diagnostic confidence in some settings.

Challenges in Histopathology Interpretation

Overlap syndromes in autoimmune liver diseases, particularly the coexistence of autoimmune hepatitis and PBC, present significant diagnostic challenges due to overlapping clinical, biochemical, immunological, and histological features [63]. Histopathological evaluation plays a crucial role in diagnosing these conditions, but its accuracy is highly dependent on the expertise of the histopathologist. Many centers lack dedicated hepatopathology expertise. This shortage can lead to suboptimal or inconsistent reporting, as general pathologists may not be equipped to identify the nuanced histological features that distinguish these conditions. In some cases, mild inflammatory changes or interface hepatitis might be misinterpreted as autoimmune hepatitis or an overlap syndrome, leading to misdiagnosis or unnecessary treatments, such as immunosuppressive therapy, rather than the appropriate treatment for PBC.

**Figure 3:**
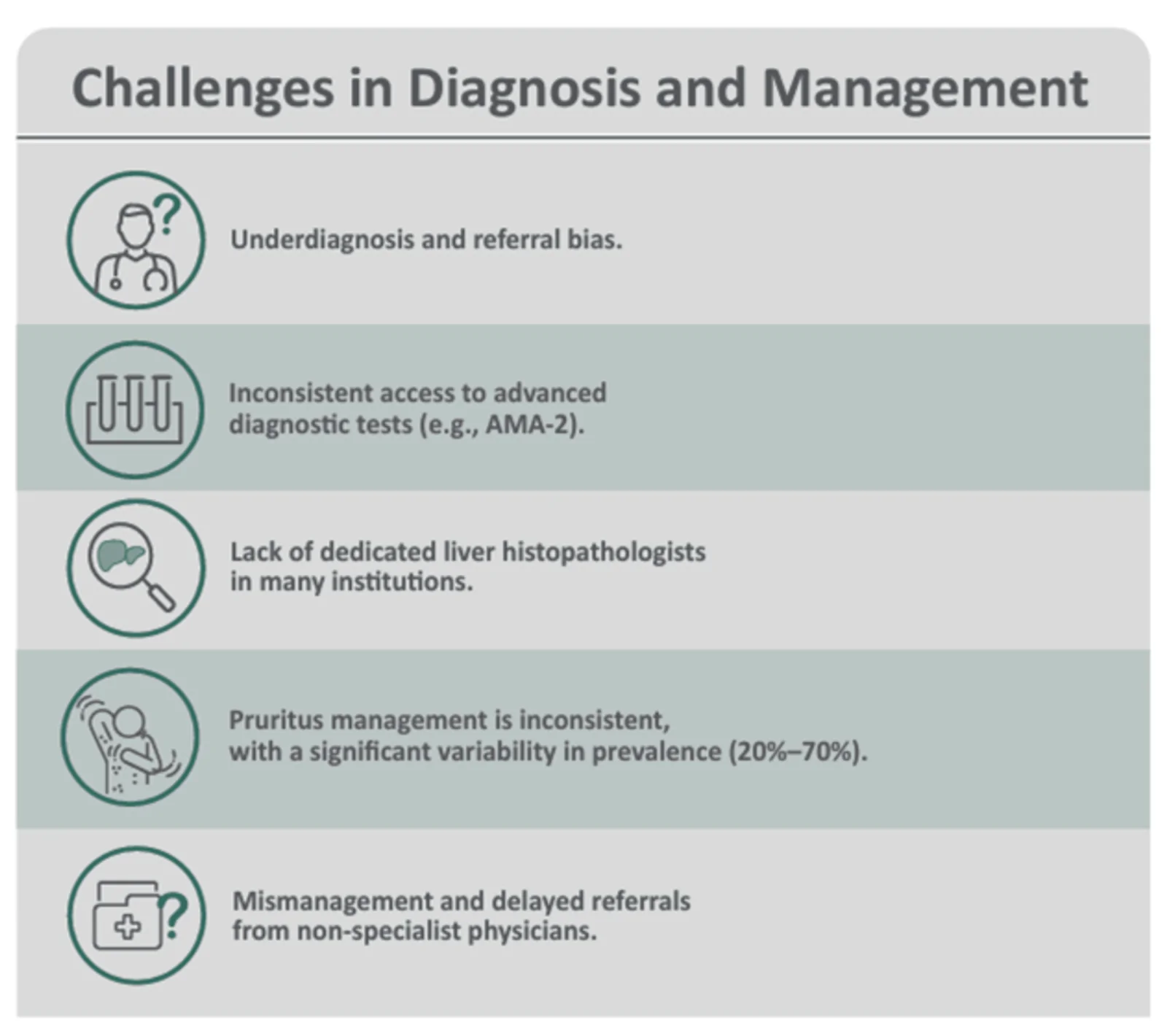
A summary of the challenges in the diagnosis and management of PBC (as per expert opinion). AMA-M2: anti-mitochondrial antibody type M2; PBC: primary biliary cholangitis. Image created by authors using Adobe Illustrator 2026 (Adobe Inc., San Jose, CA, USA).

In cases of AMA-negative patients, the accuracy of the histopathology report becomes even more crucial, underscoring the need for specialized expertise in liver pathology. A visual summary of the diagnostic and management challenges associated with PBC is provided in Figure 3.

Proposed solutions and recommendations

Improving treatment outcomes in PBC across the Middle East requires coordinated efforts to strengthen healthcare infrastructure, standardize clinical practice, and enhance professional training. Establishing national autoimmune liver disease registries, alongside prospective multicenter epidemiologic studies using standardized diagnostic criteria, is essential to accurately define disease burden, treatment patterns, and long-term outcomes, particularly in underrepresented Gulf countries.

To facilitate earlier diagnosis and reduce delays, region-specific referral guidelines for abnormal liver enzymes should be developed and targeted not only to hepatologists but also to frontline providers, such as general practitioners, endocrinologists, and dermatologists. In parallel, centralization of hepatopathology services and focused training in autoimmune liver disease could improve diagnostic consistency and biopsy interpretation.

**Figure 4:**
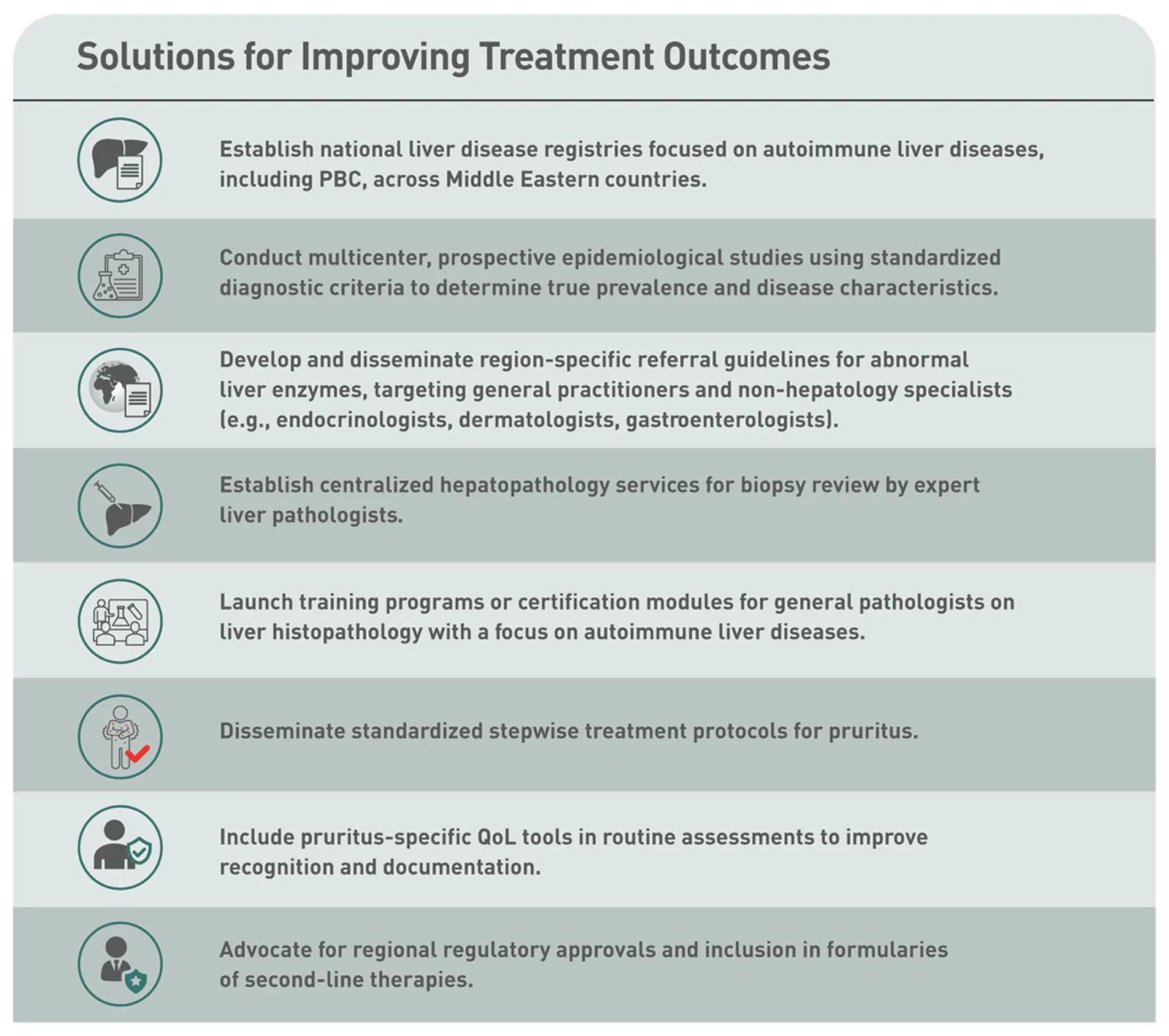
A summary of proposed solutions to improve treatment outcomes (as per expert opinion). PBC: primary biliary cholangitis; QoL: quality of life. Image created by authors using Adobe Illustrator 2026 (Adobe Inc., San Jose, CA, USA).

Standardized management pathways for pruritus, including routine use of pruritus-specific quality-of-life assessment tools, should be implemented to improve symptom recognition and documentation. Finally, advocacy for regional regulatory approval and national formulary inclusion of evidence-based second-line therapies is critical to address unmet treatment needs among UDCA non-responders. A visual summary of the proposed solutions is illustrated in Figure 4.

Limitations

This review integrates expert insights due to the limited availability of high-quality, region-specific data on PBC in the Middle East. While such clinical experience provides valuable context, it is inherently subjective and may not reflect the broader population. Prevalence estimates and treatment patterns described herein are based on the experience of hepatologists from tertiary centers, which may introduce referral bias and limit generalizability. The absence of large-scale, population-based studies remains a major gap that hinders precise epidemiological assessments and evidence-based decision-making tailored to the region.

## Conclusions

In conclusion, while awareness of PBC has grown in the Middle East, significant challenges remain. This review highlights key obstacles identified by hepatology experts in the Gulf region, particularly related to early diagnosis and limited access to essential diagnostic resources. Despite these gaps, the increasing use of UDCA and the rising interest in second-line therapies represent important progress. To improve patient outcomes, coordinated regional efforts are needed to standardize diagnostic pathways, enhance training in autoimmune liver diseases, and broaden access to advanced treatments. Establishing national clinical registries, conducting multicenter epidemiological studies, and implementing educational initiatives, particularly targeting primary care and non-hepatology specialists, will be essential. The expert insights synthesized in this review provide a pragmatic understanding of current clinical practices and underscore the urgent need for data-driven, context-specific strategies to optimize the management of PBC in the Middle East.
